# Quantifying Alignment Deviations for the In-Plane Biaxial Test System via a Shape-Optimised Cruciform Specimen

**DOI:** 10.3390/ma15144949

**Published:** 2022-07-15

**Authors:** Junxian Chen, Jianhai Zhang, Hongwei Zhao

**Affiliations:** 1 School of Mechanical and Aerospace Engineering, Jilin University, Changchun 130022, China; chenjunxianie5@gmail.com; 2 Key Laboratory of CNC Equipment Reliability, Ministry of Education, Jilin University, Changchun 130022, China; 3 Chongqing Research Institute, Jilin University, Chongqing 401120, China

**Keywords:** loading coaxiality, in-plane biaxial test, cruciform specimen, alignment deviation, automated machine learning, material testing

## Abstract

The loading coaxiality of an in-plane biaxial test system and the structure of a cruciform specimen markedly affect the test results. However, due to the lack of methods for correcting the loading coaxiality and designing the cruciform specimen, the data scatter of the test results of the in-plane biaxial test systems varies from the laboratory to different tests. To quantify the loading coaxiality of the in-plane biaxial test system, we first developed a model to calculate alignment deviations with strain distribution of the shape-optimised cruciform specimen with Automated Machine Learning (AutoML). Our results demonstrated that 99.2% (54,536 of 54,976) of the quantified errors are less than 5%. Quantifying alignment deviations for an in-plane biaxial test system has been solved. The quantified method of alignment deviations could enhance the reliability of test data, improve assembly efficiency, and aid in constructing failure criteria of materials under biaxial stress.

## 1. Introduction

Verifying the multiaxial strength theory of materials rests on reliable material test data under complex stress [1]. In-plane biaxial material testing systems are widely used to measure in-plane mechanical properties of anisotropic materials, such as sheet metal, composites, and biomaterials. The scatter in the in-plane biaxial test data varies from laboratories to tests due to the lack of unified calibration methods. Therefore, we are committed to developing and improving theories of correcting loading coaxiality and methods for designing cruciform specimens to gain reliable and accurate test data from the in-plane biaxial test system.

Biaxial tests are an emerging primary technique for characterising anisotropic, hyperplastic, and heterogeneous materials. The in-plane biaxial test investigated mechanical responses to various stress states, providing essential information for identifying the material behaviour under load by characterising the material’s local anisotropic attributes [2]. Notably, uniaxial tensile testing is the most common method for determining a material’s tensile strength. Some materials used biaxial testing. Table 1 below summarises the related standards and test methods. Note the lack of knowledge and standards related to biaxial compared to uniaxial. The main difference between these two categories is how the load is applied to the materials concerned in Refs. [3,4].

### 1.1. Boundary Shape of the Cruciform Specimen

Silva Filho (2021) and Xiao (2019) [5,6] thought of the design of cruciform specimens as the relationship between biaxial stresses that were linked directly to their geometry [5,6]. From the literature survey, they found two crucial principles to the design tasks: (a) homogeneous stress–strain distribution in the central area, enabling the slightest deviations in stress determination, and (b) avoiding deformation and stress concentrations in other areas. They discovered the fundamental geometries in the literature, paying particular attention to the two aforementioned principles [7].

**Figure 1:**
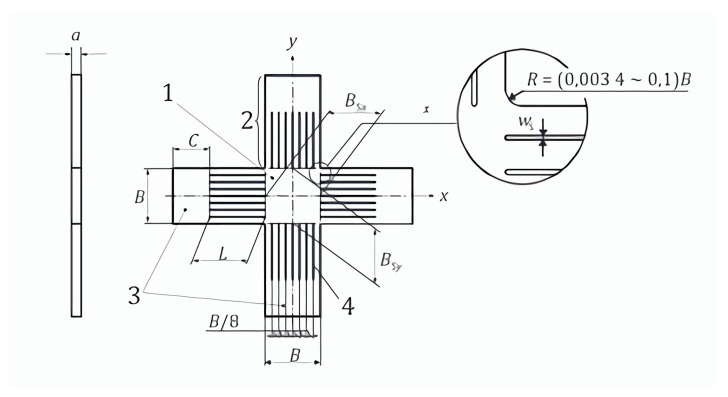
Standard shape and dimensions of the recommended cruciform test piece. Source: ISO 16842: 2021. In ISO 16842, the specimen is optimized with different objectives and different material models, enabling the specimen to be easily manufactured, the plate thickness to be constant, and the yield point to be achieved without the need for additional numerical simulations.

Cross arms of a standard cruciform specimen were often oriented at 90° and crossed over a planar gauge region. Two perpendicular axes were used to transfer the load through the grippers of the spokes. Figure 1 illustrates the cruciform specimen shape and dimensions specified in ISO 16842: 2021. Biaxial tests are typically associated with biaxial strain. Therefore, insufficient strain paths are rarely explored to construct a forming limit graph. However, by adjusting the loads’ ratio applied along the spokes, alternate stress conditions can be generated, enabling the simulation of complicated strain patterns. Precise estimation of strain fields had been a critical concern. Cruciform tests have been the best way to determine how work-hardening behaves and where the anisotropic yield points are in biaxial tension. As evidenced by the abundance of different cruciform geometry configurations in the literature, there is no other standardised geometry.

Recent work by Dhatreyi, B. (2012) [8] also discovered that two groups of parameters (cruciform shape parameters and gauge area parameters) strongly influenced the biaxial testing specimens [8]. More precisely, a fillet-shaped corner with a radius of 0.25 w was the optimal parameter, as it provided a minimum stress concentration at the intersection of the arms. From the analysis of gauge area parameters, they found a circular-shaped gauge area with a thickness ratio (tg/tarm) of 0.1 and with the largest possible transition radius (rf) to be optimal, as it improved the state of stress in the gauge area. The coupling of these optimum parameters was an excellent interaction when the gauge area size (a) was equal to 0.6 times the arm’s width (w). Using these dimensions, biaxial specimens were fabricated by a simple hand layup technique at room temperature and subjected to equibiaxial loads using an in-plane biaxial test rig. These cruciform specimens were found to fail along the line, making an angle of 45° with the principal loading axis, which confirmed the prediction of simulations that raised stresses governed the failure initiation near the transition zone rather than the biaxial stresses in the gauge area.

Turning to the perspective of Creuziger, A. (2017) [9], the researchers found that the fillet diameter was critical in cruciform geometry—A significant challenge to fabricating built-up specimens that were made without thinning the centre of the sample. Another insight they discovered was that different mechanisms that included stress near the fillet and strain near the notches constrained the deformation of the sample at different places. These constraints, such as the size and extent of diffuse, are currently challenging to model accurately.

### 1.2. Misalignments of the In-Plane Biaxial Test System

**Figure 2:**
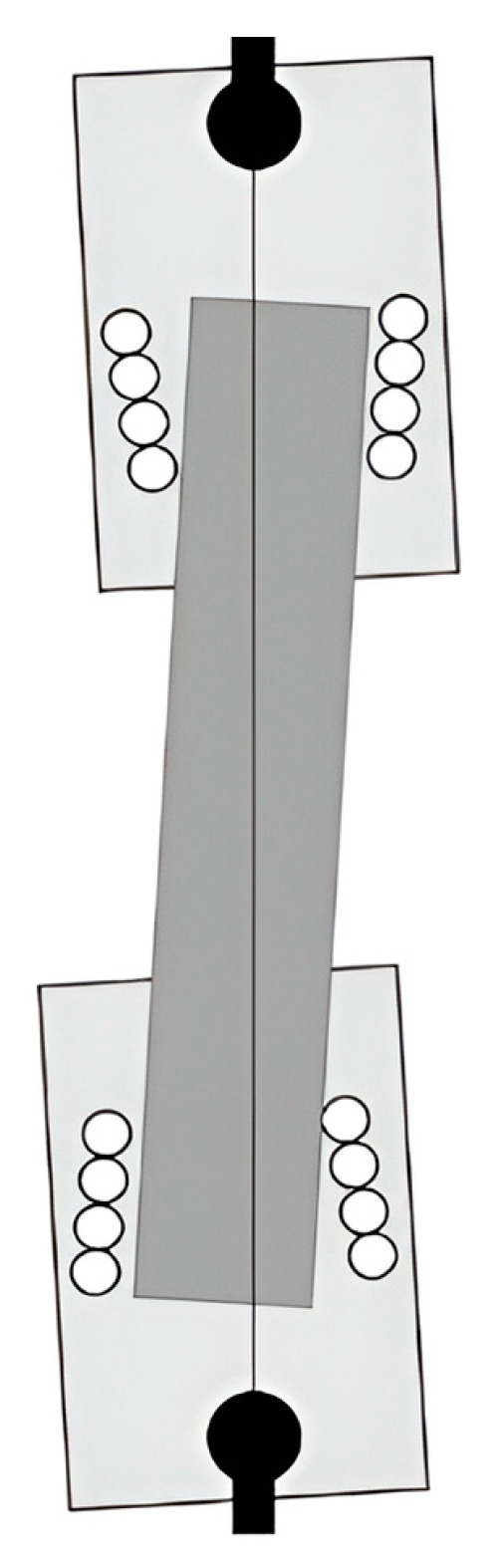
Specimen misaligned within the aligned system. The “misalignment” is defined as a linear or angular deviation of the central axis between two fixtures.

The term “alignment” can be defined as the condition of a testing machine that influences the introduction of bending moments into a specimen (or alignment transducer) during the application of tensile or compressive forces, as specified in ASTM E1012 (2020) (Figure 2).

Uniaxial alignment was well described (High-Temperature Mechanical Testing Committee (HTMTC) [10]; 1998, Kandil, F.A. [11,12,13]). There was, however, a deficiency of knowledge regarding methods of correcting the loading coaxiality and designing the cruciform specimen. Pini, L. (2020) [14] observed misalignments related to the three different coordinate systems implied in the literature and demonstrated that all the misalignment is assumed to be due to the unperfect clamping of the specimen within the plate [14].

The loading coaxiality of the biaxial material test system markedly affects the test results of the materials. Similar to the uniaxial test system [15,16], poor alignment of the test system introduces additional bending stress that aggravates the stress concentration of the cruciform specimen at the interaction and impacts the reliability of the test results [5]. Compared with the uniaxial test system, the in-plane biaxial test system has higher requirements of loading coaxiality [17]: the same loading direction and coplanarity of two mutually perpendicular loading axes. However, current standards cannot quantify the alignment deviations of the biaxial test system because the uniaxial alignment adjustment procedure considers only the effect of the alignment error on a single axis [18]. Given the alignment strategy of the uniaxial material test system, we could quantify the alignment deviations with the strain distribution of an appropriate cruciform specimen.

### 1.3. Quantifying Alignment Deviations

The cruciform specimen’s structure significantly affects the strain distribution at the interaction of the cross-arms. Poor cruciform specimen structure leads to premature failure because of stress concentration at cross-arms interaction [19,20]. Many scholars have optimised the boundary shape of cruciform specimens to prevent premature failure with various methods [21,22,23]. However, because of cruciform specimens’ high sensitivity to geometries, current studies cannot reach a consensus on specimens’ boundary shape [24,25]. Hence, we should develop new methods to design the specimen for the in-plane biaxial test.

Although numerous institutions have developed various in-plane biaxial test systems, including vertical biaxial test systems [23,26], horizontal biaxial test systems [27,28,29,30,31], and others equipped with high-temperature furnaces [30], there is currently no practical method for assessing alignment deviations of an in-plane biaxial testing system, and no test has been conducted to determine the effect of alignment deviations on test results. Notably, integrating high-temperature furnaces to the in-biaxial tensile test system could aggravate alignment issues due to the extension of the loading chain. As a result, the problem of quantifying alignment deviations of the in-plane biaxial tensile testing system is unclear and urgently needs to be solved.

This work aims to quantify alignment deviations of the in-plane biaxial test system. Through AutoML, we determined a relationship between alignment deviations and strain measurement points to quantify alignment deviations. Utilising the least square method, we proposed identification coefficients to quantify the contribution of each alignment deviation to strain distribution. Our results demonstrated that 99.2% (54,536 of 54,976) of the quantified errors of alignment deviations are less than 5%, and the size of each alignment deviation positively correlated with the corresponding identification coefficient. Quantifying alignment deviations for an in-plane biaxial test system via a shape-optimised cruciform specimen has been solved. In addition, a new boundary form for cruciform specimens has been proposed based on nonparametric shape optimisation. The quantified model may enhance the reliability of the test data and improve assembly efficiency.

## 2. Materials and Methods

### 2.1. Shape Optimization Model

**Figure 3:**
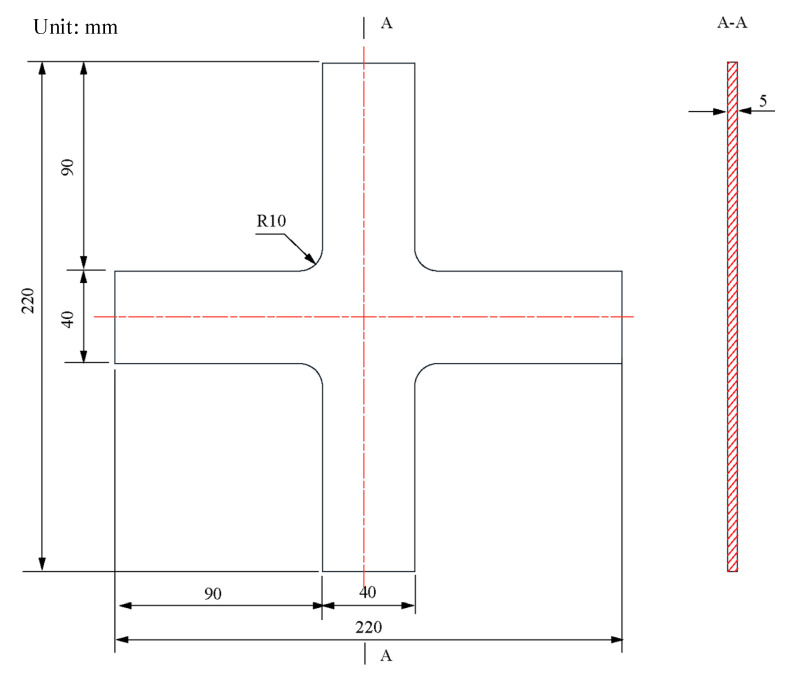
The classic structure of the cruciform specimen. The structure of the cruciform specimen serves as the initial condition for optimisation.

The following are the five primary dimensions of the classic cruciform specimen: the length is 220 mm; the width is 220 mm; the thickness is 5 mm; the cross-arms width is 40 mm, and the fillet at the corner is 10 mm (Figure 3).

**Figure 4:**
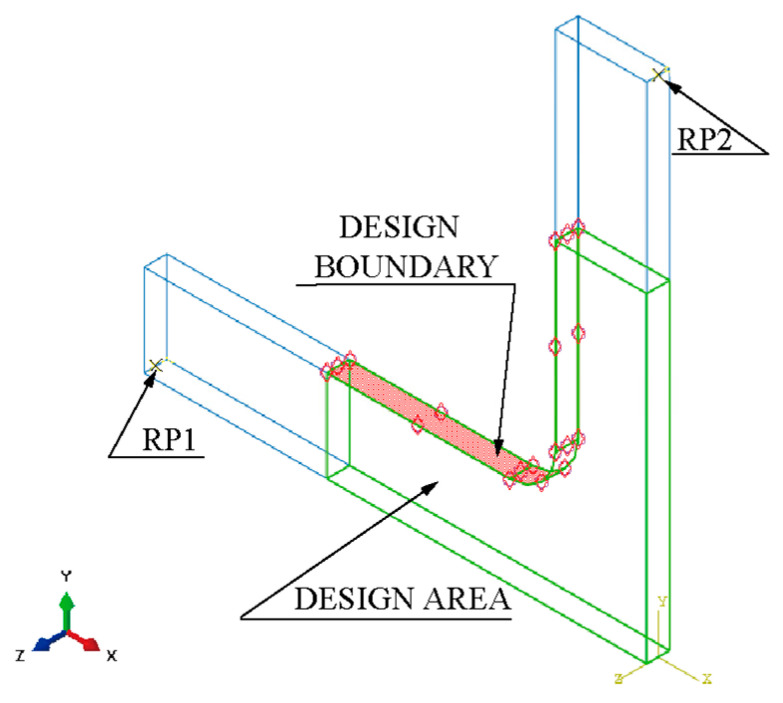
Physical model of a cruciform specimen for shape optimisation.

Resting on the symmetries of the geometry and force boundary conditions about the X and Y axes, a quarter model of the classic structure was adopted (Figure 4). The quarter model comprises four parts: clamping part X1, part X2, design boundary, and design area.

We employed the shape optimisation method—one nonparametric method—to minimise the stress concentration at the specimen’s corner [32].

**Figure 5:**
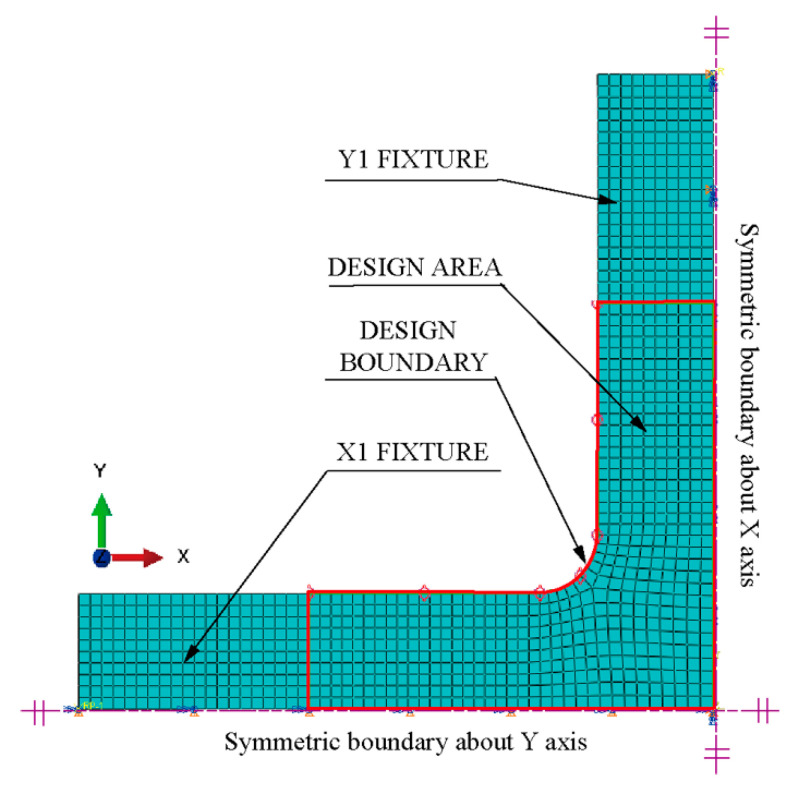
Simulation model of the cruciform specimen for shape optimisation.

The finite element model consists of the following (Figure 5):

1. Parts: The model consists of a single part built according to Figure 3 and Figure 4.
2. Mesh: The type of hexahedral element is C3D8R, and the total number of nodes and elements is 4452 and 3003, respectively (Figure 5). The five-element layers were arranged along the *Z*-axis (the thickness direction).
3. Materials: The Young’s modulus, yield strength, and Poisson’s ratio of HC1200 are 210 GPa, 1200 MPa, and 0.3, respectively.
4. Steps: Only one step is specified. Nonlinear geometric effects are considered.
5. Loads: We rigidly coupled the clamping parts X1 and X2 with two reference points, RP1 and RP2 (Figure 4), respectively, to apply distributed force on the clamping parts with the concentrated forces on RP1 and RP2. One load of magnitude 40 kN is specified in RP1 and RP2.
6. Boundary conditions: Symmetry boundary conditions are applied to specified regions (Figure 5). Before proceeding with the optimisation analysis, examine the finite element model.

**Figure 6:**
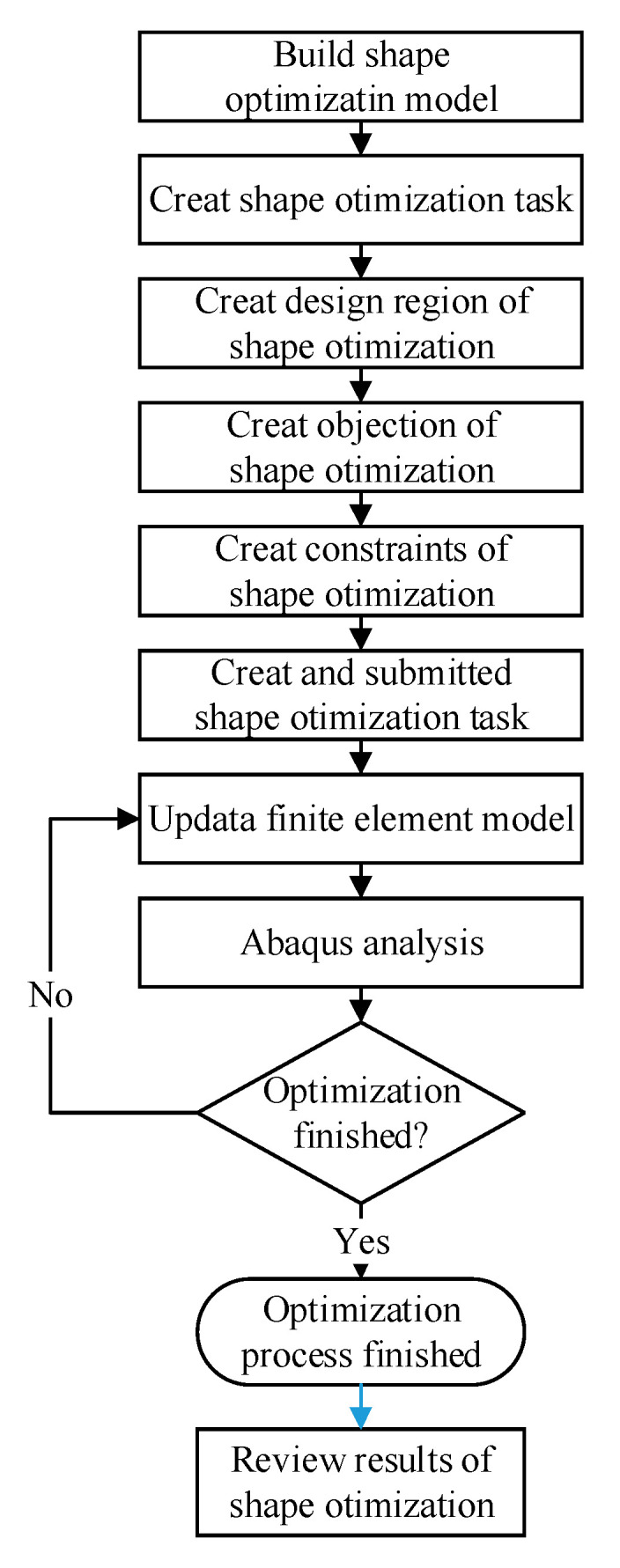
The shape optimised processes for the classic cruciform specimen.

Configuring a shape optimisation analysis using Abaqus (Figure 6):

1. Creating an optimisation task:
  - Switch to the Optimisation module. In the Create Optimization Task dialogue box, select the set DESIGN BOUNDARY, as shown in Figure 5.
  - In the Basic tabbed page, select Freeze boundary condition regions: the nodes clamped by the X1 fixture and X2 fixture; the nodes on the symmetry edges to the symmetrical planes (Figure 5).
  - Select Specify smoothing region and select the whole model.
  - Select Fix all as the number of node layers adjoining the task region to remain free.
  - In the Mesh Smoothing Quality tabbed page, set the Target mesh quality to Medium.
2. Creating design responses:
  - In the Create Design Response dialogue box: Accept Single-term as the type, and select Whole Model as the design response region.
  - In the Edit Design Response dialogue box, select Stress and Mises hypothesis (The field Operator on values across steps and load cases is set to Maximum value by default).
3. Creating an objective function: In the Edit Objective Function dialogue box, add all design responses eligible to participate in an objective function and change the Target to Minimise the maximum design response values (Equation (1)).
4. Creating a constraint: Select Volum for the Design Response, Toggle on A fraction of the initial value and enter 1 (Equation (2)).
5. Submit an optimisation process for the optimisation task optimise-shape. Note that the Maximum cycles field is set to 50 by default for shape optimisation.

Typically, in shape optimisation, the goal is to homogenise the stress on the surface of a component by adjusting the surface nodes (moving them inward or outward). Thus, minimisation is achieved by homogenisation. Shape optimisation is not limited to minimising stresses; it may be extended to plastic strains, natural frequencies, etc. This research will homogenise the Mises stress on the cross-arms intersection. The following is the objective of shape optimisation:

(1) $$Min:\sigma_{von\_mises},$$

where $\sigma_{von\_mises}$ is the Mises stress of each node in the design boundary (Figure 4).

The purpose of creating volume constraints in shape optimisation is to ensure that the overall volume of the component remains the same. In some cases, adding material to reduce stress might be undesirable. In such cases, we can redistribute the material to minimise the stress. Volume constraints ensure that either no material is added or very little material is added due to the shape optimisation. The following is the volume constraint of shape optimisation:

(2) $$V_{\mathrm{final}}=V_{\mathrm{initial}\;},$$

where *V*_initial_—is the initial volume of all elements;

*V*_final_—the final volume of all elements.

### 2.2. Quantified Model of Alignment Deviations

AutoML was used to map the relationship between the alignment deviations and the strain measurement points using the optimised specimen. The training data were obtained using FEM via optimum Latin hypercube Latin (opt LHD) [32].

The ideal clamping state yields that the alignment deviations—originating from the spatial dislocation of the central axis of the fixtures—are independent of the geometries of the cruciform specimen. The additional bending strain comes from two aspects: the dislocation of the central axis between the fixtures in space and the unsatisfactory clamping state of the fixture to the specimen. The unsatisfactory clamping state of the fixture to the specimen leads to an eccentric load, which varies with the cruciform specimen’s geometries, such as the length, width, and thickness. Theoretically, the dislocation of the central axis between fixtures in space is the attribute of the test system itself, independent of the geometry of the cruciform specimen. Resting on the ideal clamping state of the fixture, we only consider alignment deviations: the spatial dislocation of the central axis between fixtures.

**Figure 7:**
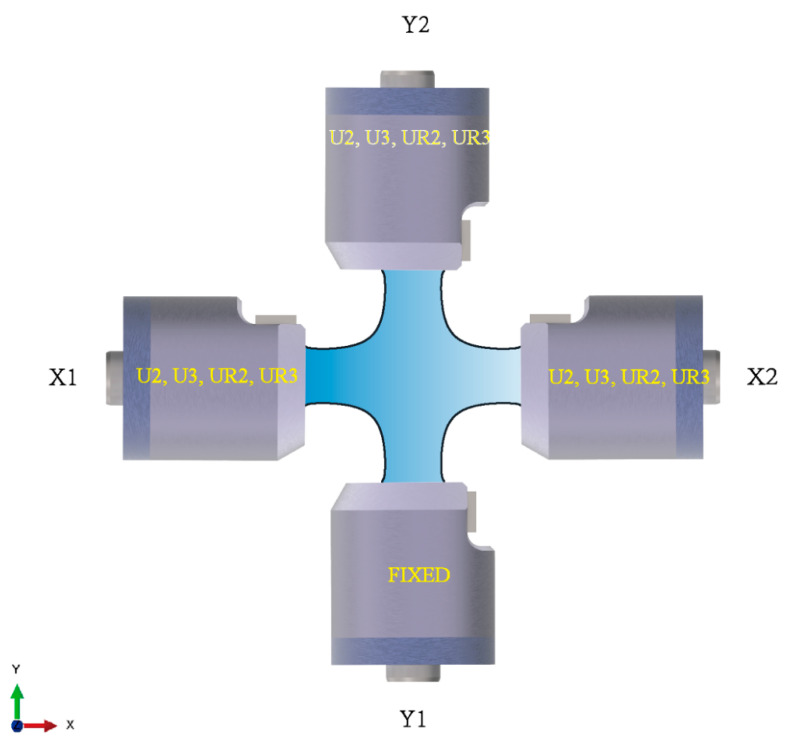
Alignment deviations of the in-plane biaxial test system.

The displacement and coordinate system provisions are identical to those in ABAQUS: Linear displacement along the X, Y, and Z axes are denoted by U2, U2, and U3, while angular displacement along the X, Y, and Z axes are denoted by UR2, UR2, and UR3, respectively (Figure 7). To map the quantified model of alignment deviations, we picked 12 alignment deviations from the in-plane biaxial test system (Figure 7), including four alignment deviations (U2, U3, UR2, UR3) of X1, four alignment deviations (U2, U3, UR2, UR3) of X2, and four alignment deviations (U2, U3, UR2, UR3) of Y1.

The generality of the hydraulic and profiling fixtures and 12 alignment deviations is maintained without losing generality. The fixtures of hydraulic and profiling fixtures markedly differ in clamping form. The six alignment deviations (U2 and UR3 in X1; U2 and UR3 in X2; U2 and UR3 in Y1) cannot exist for the hydraulic fixtures. The 12 alignment deviations involved in X1, X2, and Y1 are inevitable for the profiling fixtures because of the limitation of locators/clamps, such as grooves. The strain distribution on the specimen depends on the constraints of the fixture. The constraints of the six alignment deviations cannot exist for the hydraulic fixtures, indicating that the contribution to the strain distribution must be close to zero. Hence, the working condition—the cruciform specimen clamped by hydraulic fixtures—is only a particular case in this study.

**Figure 8:**
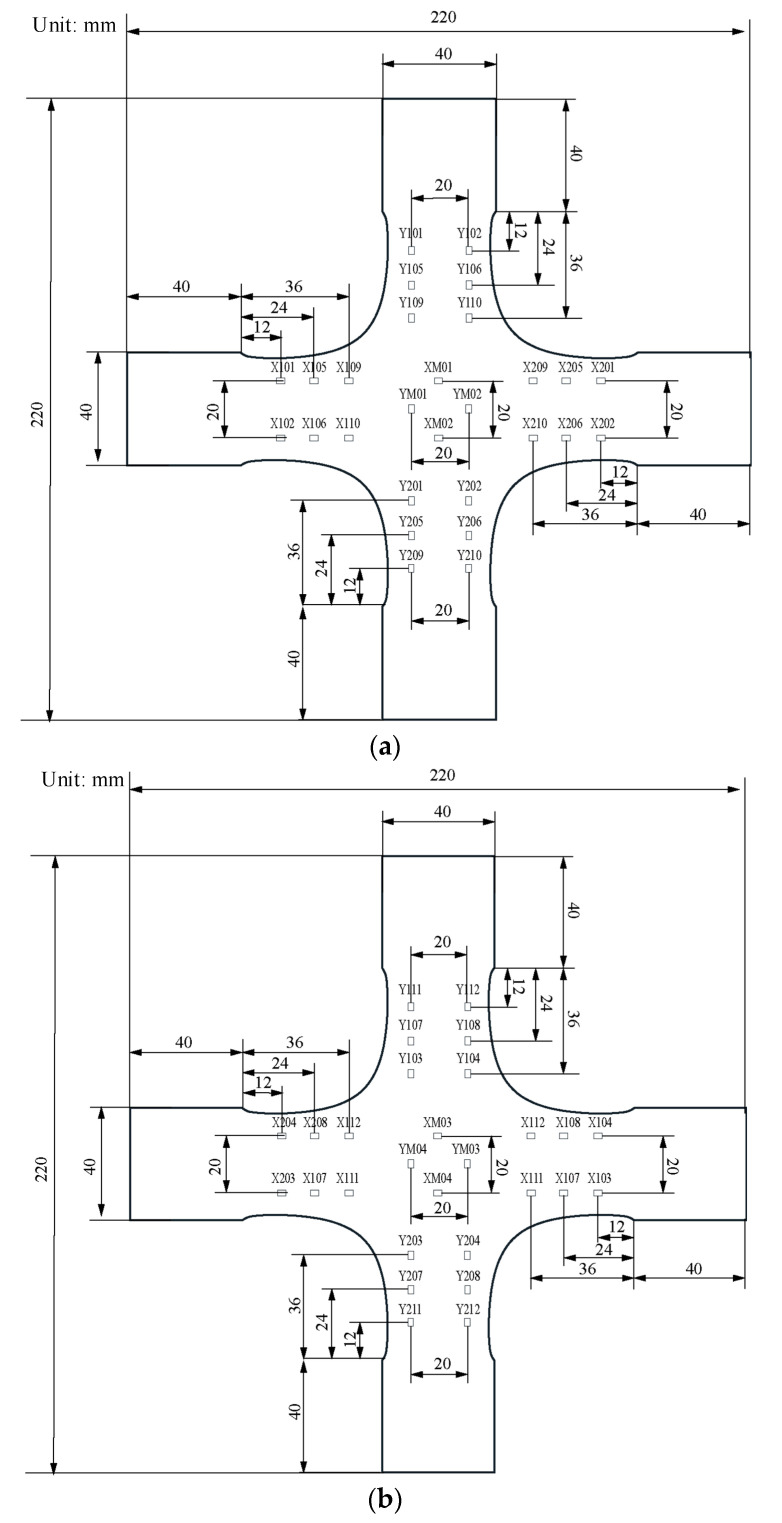
The distribution diagram of 56 strain measurement points. (**a**) The front face of the optimised cruciform. (**b**) The back of the optimised cruciform.

Fifty-six strain points were applied to quantify alignment deviations of the in-plan biaxial test system. Fifty-six strain points include 12 strain points on each arm to measure the coaxiality of the corresponding loading chain and eight measurement points in the centre to measure the interaction of the four loading chains (Figure 8).

**Figure 9:**
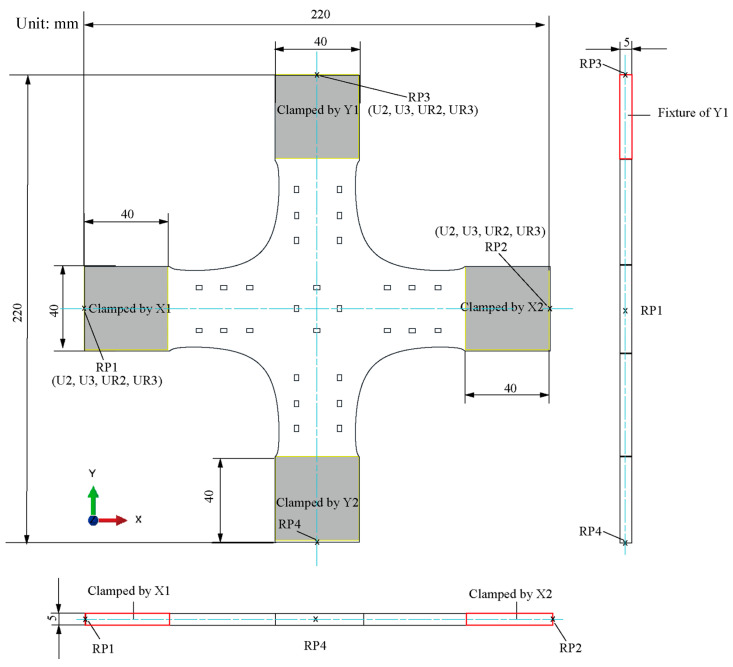
Physical model of the cruciform specimen.

The first clamping end of the cruciform specimen determines the coordinate system of the physical model. Y2 is assumed to be the first clamping end, and its coordinate system corresponds with the global coordinate system (Figure 9). In the simulation process, the following three settings have been made: freeze the six degrees of freedom of the Y2 end; free X1U2, X2U2, and Y1U2; couple the four clamping ends with the reference points: RP1, RP2, RP3, and RP4, respectively. In addition, to simulate different alignment deviations, we change the displacement boundary conditions with four reference points. 

**Figure 10:**
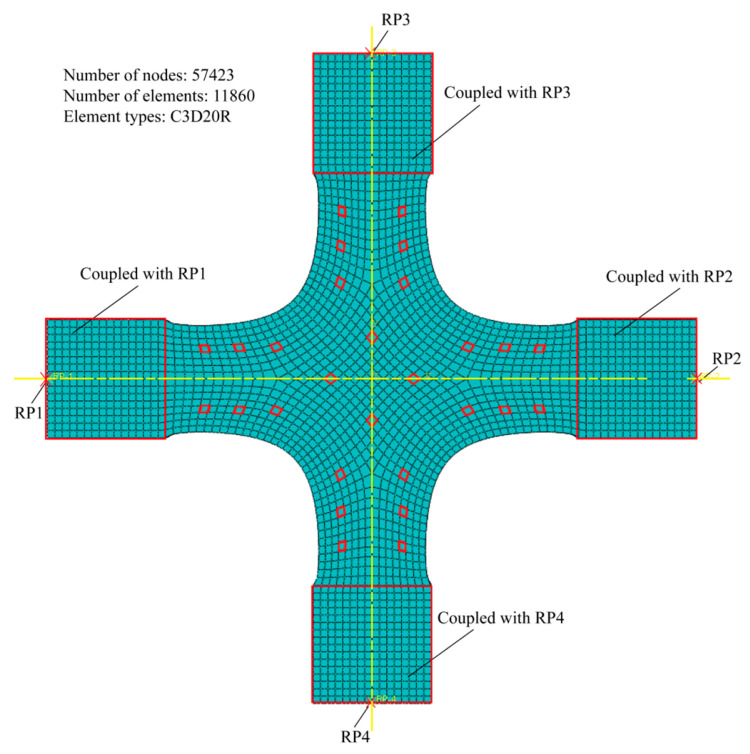
Finite element model of cruciform specimens.

A static analysis module built into ABAQUS was employed to calculate the strain values from 56 measurement points (Figure 10). The analysis were conducted by Abaqus version 2019 for Windows (Dassault Systemes Simulia Corp., Johnston, RI, USA). For each simulation, the input and output of the finite element model are 12 alignment deviations and 56 strain values, respectively. The following is the specific information about the finite element model: The material of the specimen adopts HC1200, whose elastic modulus, yield strength, and Poisson’s ratio of HC1200 are 210 GPa, 1200 MPa, and 0.3, respectively; the finite element type is linear hexahedral elements C3D20R, and the maximum size of mesh generation is 1 mm. The specimen comprises 11,860 elements and 57,423 nodes by automatic mesh generation technology. We highlight that the material deformation should be limited to the elastic range [9], so we only list the elastic modulus, yield strength, and Poisson’s ratio.

According to the alignment deviations through the Opt LHD, we performed 54,976 finite element simulations (Table 2). We extracted the 56 strain values corresponding to each group of alignment deviations for each simulation by writing a secondary development script.

The AutoML built-in AutoGloun was utilised to quantify alignment deviations of the in-plane biaxial test system using the 54,976 training data [33]. In contrast to the finite element model, the input variables of the AutoML model are strain values of 56 measurement points, and the output variables are 12 alignment deviations (Figure 7). The data were analyzed by AutoGLuon version 0.5.0 for Windows (Author: AutoGluon Community). The AutoGluon will repeatedly modify structural parameters, increase training times, or increase the train number to gain quantified alignment deviations. The training process stops, and the model is stored when the training time reaches the preset value.

### 2.3. Identification Coefficients of Strain Distribution

The least-squares method (LMS) was used to quantify each alignment deviation’s contribution to the specimen’s strain distribution. Accordingly, we defined each alignment deviation as a mode of characteristic strain. Hence, the 12 alignment deviation yields 12 modes of characteristic strain. The above 12 characteristic modes can linearly express any strain distribution determined by 12 alignment deviations.

To quantify the contribution of the alignment deviation to the strain distribution of the specimen, we normalise each characteristic strain mode under the same rule:

(3) $$s_{ij}=s_{ij}^{*}/\sqrt{\sum_{i=1}^{56}{\left(s_{ij}^{*}\right)}^{2}}(I=0,1,2,3,...12),$$

where $s_{ij}$—the normalised strain of the *j*-th alignment deviation at the *i*-th strain measurement position;

$s_{ij}^{*}$—the strain value of the finite element simulation of the *j*-th alignment deviation at the *i*-th strain measurement position.

With the combined action of the above 12 alignment deviations, the 56 strain values on measurement points can be expressed as:

(4) $$S^{*}=\left[\begin{array}{ccccc} e_{0} & e_{1}s_{11} & e_{2}s_{12} & e_{3}s_{13} & \ldots \\ e_{0} & e_{1}s_{21} & e_{2}s_{22} & e_{3}s_{23} & \ldots \\ e_{0} & e_{1}s_{31} & e_{2}s_{32} & e_{3}s_{33} & \cdots \\ \vdots & \vdots & \vdots & \vdots & \vdots \\ e_{0} & e_{1}s_{n1} & e_{2}s_{n2} & e_{3}s_{n3} & \ldots \end{array}\begin{array}{c} e_{12}s_{1m}\\ e_{12}s_{2m}\\ e_{12}s_{3m}\\ \vdots \\ e_{12}s_{nm} \end{array}\right],$$

where ***S*** * is the matrix with 56 strains on measurement points. In formula (4), *n* is 56, *m* is 12, and *e*_0_ represents the constant strain component caused by the test force.

The following is the matrix form of linear Equation (4):

(5) $$S^{*}=Se,$$

where ***S***—the *i*-th characterisation of strain distribution;

***e***—the *i*-th characteristic strain identification coefficients.

The problem of solving ***e*** in Formula (5) by the least square method can be expressed as:

(6) $$\underset{e}{\min}{\left\|Se-S^{*}\right\|}_{2},$$

Therefore, the following solves Formula (6):

(7) $$e={\left(S^{T}S\right)}^{-1}S^{T}S,$$

To verify the linear independence of the 12 modes of characteristic strain, we calculate the rank of the matrix. The rank p of the above matrix can be expressed as:

(8) $$p=rank\left(\left[\begin{array}{cccc} s_{11} & s_{12} & \ldots & s_{1m}\\ s_{21} & s_{12} & \cdots & s_{2m}\\ \vdots & \vdots & \vdots & \vdots \\ s_{n1} & s_{12} & \ldots & s_{nm} \end{array}\right]\right),$$

The validity of the identification coefficient of zero alignment deviation was verified through two control groups. In nine cases, we set four alignment deviations of Y1 zero compared with nonzero alignment deviations involved in X1 and X2. In the cases of AD-1 to AD-8, we set alignment deviations—U2, U3, UR2, UR3 of X1 and the four U2, U3, UR2, and UR3 of X2—to zero compared with nonzero alignment deviation in the initial. In addition, to verify the numerical validity of the identification coefficients, we set X1 (U2, U3, UR2, UR3) and X2 (U2, U3, UR2, UR3) as the experimental control group because of numerical comparability originating from geometric symmetry (Table 3).

## 3. Results

### 3.1. Predictive Accuracy of Alignment Deviations

This optimal quantified model of 12 alignment deviations was weightedEnsemble_L3, a weighted three-layer stacking model built-in AutoGluon [33]. To analyse the accuracy of the alignment deviations, we define the quantified error as *E* and the percentage of the quantified error as *P*.

The following expresses *E*:

*E* = *A_p_* − *A_f_*, (9)

where *A_p_*—the alignment deviation quantified by AutoML;

*A_f_*—the alignment deviation used for FEM simulation with the same strain distribution of *A_p_*.

The following expresses *P*:

*P* = Abs (*E*/*S*) × 100%, (10)

where *S*—the variation range of the alignment deviation, corresponding to *E*, shown in Table 2.

To assess the validity of the quantified error *E*, we used error analysis. By constructing a Python3 script file, we analysed the data (Table 3). The mean values of the quantified error of the alignment are concentrated around zero according to the analysis of the confidence interval of the mean value with a confidence level of 99.9%. The means of the quantified errors of U2, U3, UR2, and UR3 of X1 are −4.20 × 10^−5^ (99.9% CI, −7.2 × 10^−5^ to −1.20 × 10^−5^), −3.10 × 10^−5^ (99.9% CI, −1.06 × 10^−4^ to −4.40 × 10^−5^), 1.20 × 10^−5^ (99.9% CI, −9.80 × 10^−8^ to 1.22 × 10^−4^), and 8.30 × 10^−5^ (99.9% CI, 0 to 1.66 × 10^−4^), respectively. The mean prediction errors of U2, U3, UR2, and UR3 of X2 are 8.23 × 10−5 (99.9% CI, 1.73 × 10−5 to 1.77 × 10^−4^), −8.92 × 10^−4^ (99.9% CI, −2.29 × 10^−4^ to 5.08 × 10^−5^), 6.80 × 10^−5^ (99.9% CI, −4.20 × 10^−5^ to 1.78 × 10^−4^), and −3.10 × 10^−5^ (99.9% CI, −1.13 × 10^−4^ to 5.10 × 10^−4^), respectively. The mean prediction errors of U2, U3, UR2, and UR3 of Y1 are 4.30 × 10^−5^ (99.9% CI, −1.40 × 10^−5^ to 1.00 × 10^−4^), −8.70 × 10^−5^ (99.9% CI, −1.57 × 10^−4^ to −1.70 × 10^−5^), −4.00 × 10^−5^ (99.9% CI, −1.34 × 10^−4^ to 5.40 × 10^−5^), and −6.00 × 10^−5^ (99.9% CI, 3.00 × 10^−6^ to 1.17 × 10^−6^), respectively. In addition, out of the 54,976 groups of the percentage of all the prediction errors (U2, U3, UR2, UR3), 54,536 (99.2%) are less than 5%. Therefore, 99.2% of the quantified value falls centred on zero and bounded by the percentage of the quantified error of 5%.

Correlation analysis was used to evaluate the accuracy of the approximations between the quantified data and finite element simulations. The goodness-of-fit R2 and Pearson-R between the quantified data and finite element analysis models exceeded 0.999 (Table 4).

The statistical analysis was employed to verify the statistical distribution of experimental points designed by Opt LHD (Table 5). Twelve one-sample Kolmogorov–Smirnov tests were conducted to evaluate the uniformity of the distribution of discrete variables. Asymptotic significances are displayed with a significance level of 0.05. The distributions of X1 (U2, U3), X2 (U2, U3), and Y1 (U2, U3) are uniform, with a minimum of −1.00 mm and a maximum of 1.00 mm (95% CI, −0.011 to 0.011). The distributions of X1 (UR2, UR3), X2 (UR2, UR3), and Y1 (UR2, UR3) are uniform, with a minimum of −0.0122 rad and a maximum of 0.0122 rad (95% CI, −0.0001 to 0.0001).

### 3.2. Characterization of Strain Distribution with Specific Deviation

**Figure 11:**
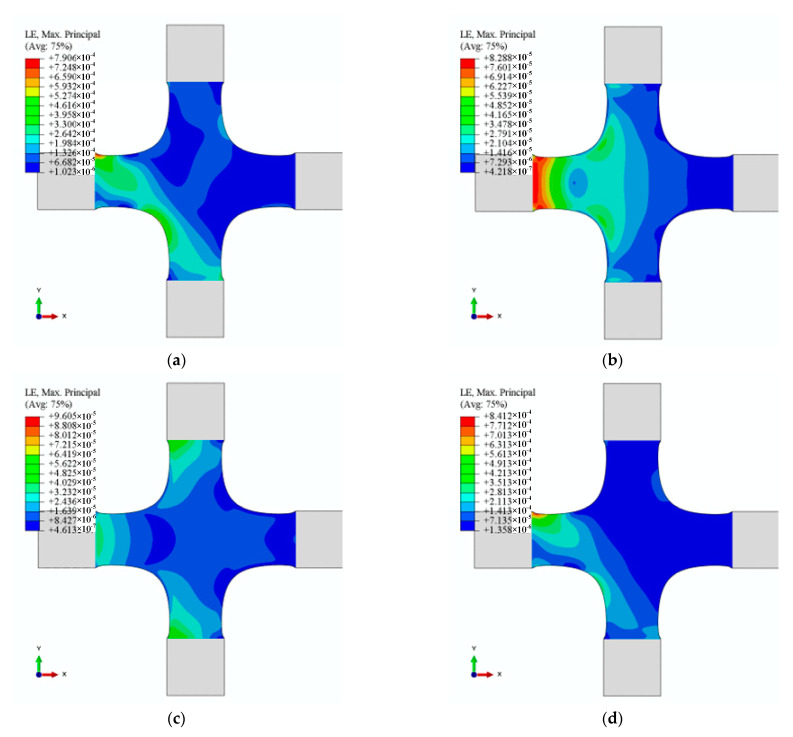
Strains distribution determined by X1. (**a**) Strain distribution with U2 (0.05 mm), (**b**) strain distribution with U3 (0.05 mm), (**c**) strain distribution with UR2 (0.0006 rad), and (**d**) strain distribution with UR3 (0.0006 rad).

**Figure 12:**
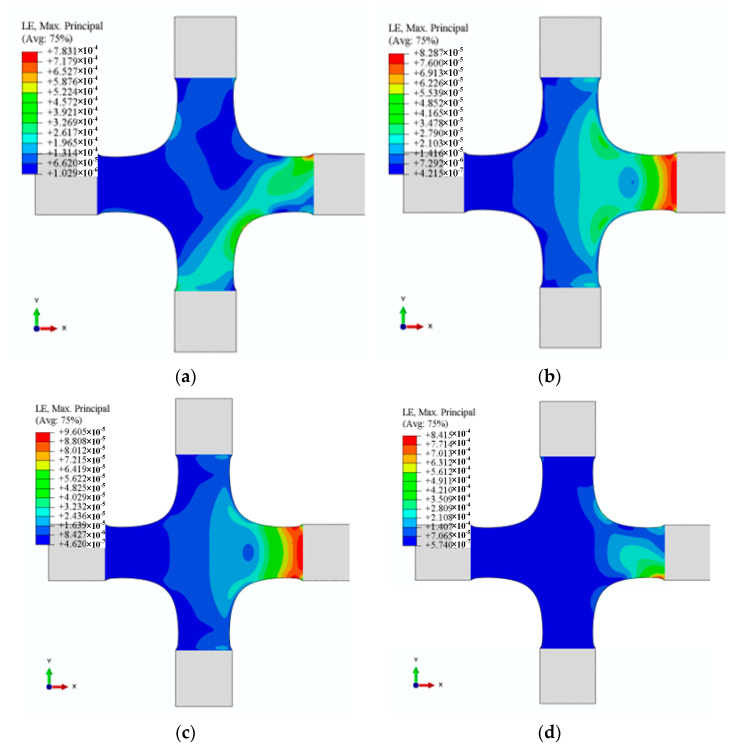
Strains distribution determined by X2. (**a**) Strain distribution with U2 (0.05 mm), (**b**) strain distribution with U3 (0.05 mm), (**c**) strain distribution with UR2 (0.0006 rad), and (**d**) strain distribution with UR3 (0.0006 rad).

**Figure 13:**
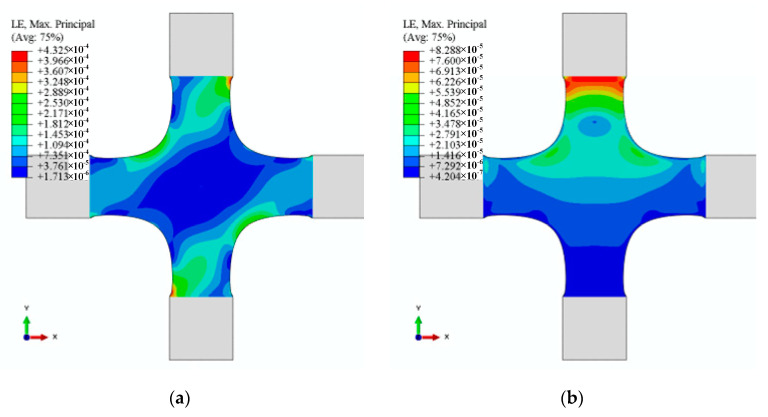

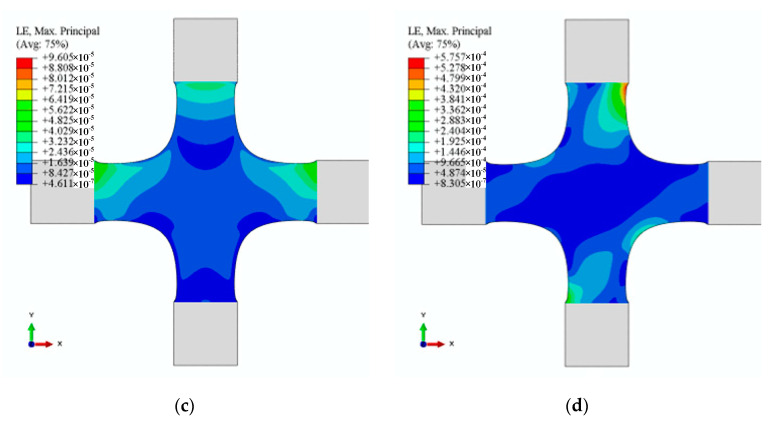
Strain distribution determined by Y1. (**a**) Strain distribution with U1 (0.05 mm), (**b**) strain distribution with U3 (0.05 mm), (**c**) strain distribution with UR1 (0.0006 rad), and (**d**) strain distribution with UR3 (0.0006 rad).

To verify the linear independence of 12 characteristic strain modes, we simulated the cruciform specimen’s strain distribution under 12 alignment deviations (Figure 11, Figure 12 and Figure 13). The results demonstrated that the characteristic strain mode corresponds to the alignment deviation. However, Figure 11, Figure 12 and Figure 13 demonstrated that each pair—U2/UR3 and U3/UR2 in X1, U2/UR3 and U3/UR2 in X2, and U2/U3 and UR2/UR3 in Y1—exhibit similarity in strain distribution.

The rank of the matrix—composed of 12 characteristic strain vectors—is 12, which implies linear independence of the 12 characteristic strain vectors. Despite linear independence, the non-orthogonality of 12 vectors incurs interaction between vectors. From the correlation coefficient matrix, the absolute correlation coefficients of X1U2 and X1UR3, X1U3 and X1UR2, X2U2 and X2UR3, X2U3 and X2UR2, Y1U2 and Y1UR3, Y1U3 and Y1UR2 are 0.677, 0.783, 0.683, 0.791, 0.661, and 0.790, respectively (Table 6). The other correlation coefficients are relatively small compared with the above six pairs.

The identification coefficients could quantify each alignment deviation’s contribution to the specimen’s strain distribution using the least square method. The following two control groups imply the validity of identification coefficients of zero alignment deviation: Among nine cases, no corresponding strain components with loads, and the alignment deviations of Y1 yield e_0_ and e_9_–e_12_ close to zero; in each case of AD-1 to AD-8, the alignment deviation of X1 and X2 set to zero leads to the corresponding strain components close to zero (Table 7). In addition, the following control group indicates numerical validity of the identification coefficients to the contribution of the alignment deviation to the strain distribution: the alignment deviations of X1U2/X2U2, X1U3/X2U3, X1UR2/X2UR2, and X1UR3/X2UR3 correspond to the identification coefficients in proportion.

### 3.3. Optimized Cruciform Specimen

**Figure 14:**
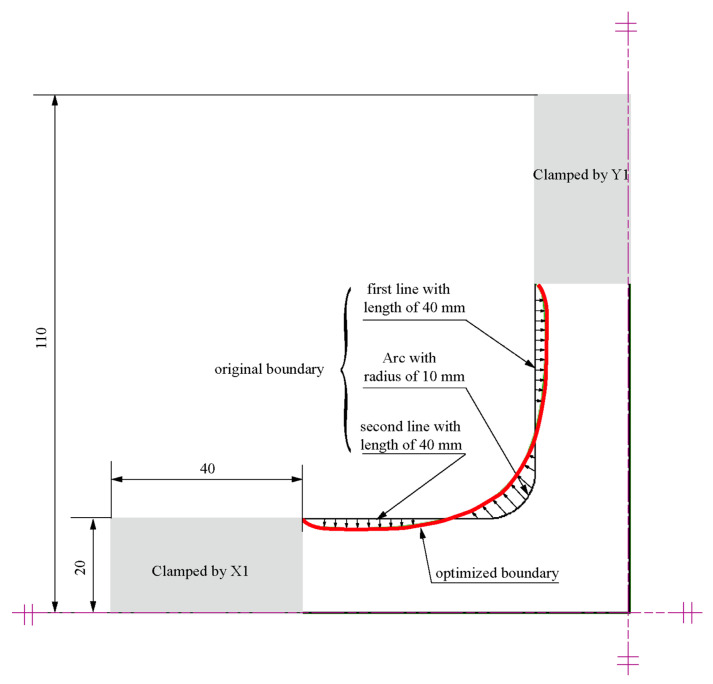
Comparison of the original boundary and optimised boundary.

Shape optimisation rests on the position change of the boundary nodes to optimise the maximum stress on the boundary (Figure 14). The boundary of the original specimen comprises two straight lines of 40 mm and an arc of 10 mm. Compared with the original specimen, shape optimisation reflects the topological characteristics: the nodes in the straight section retract inward, and the nodes at the fillet extend outward.

We used Equation (10) to fit the boundary shape based on the optimised shape boundary in Figure 14.

(11) $$y=a/(1+be^{-cx}),$$

The parameters *a*, *b* and *c* in Equation (10) were solved using 31 points along the shape-optimised curve. The solution results are shown in Table 8. The coefficient of determination (goodness of fit) exceeds 0.99.

**Figure 15:**
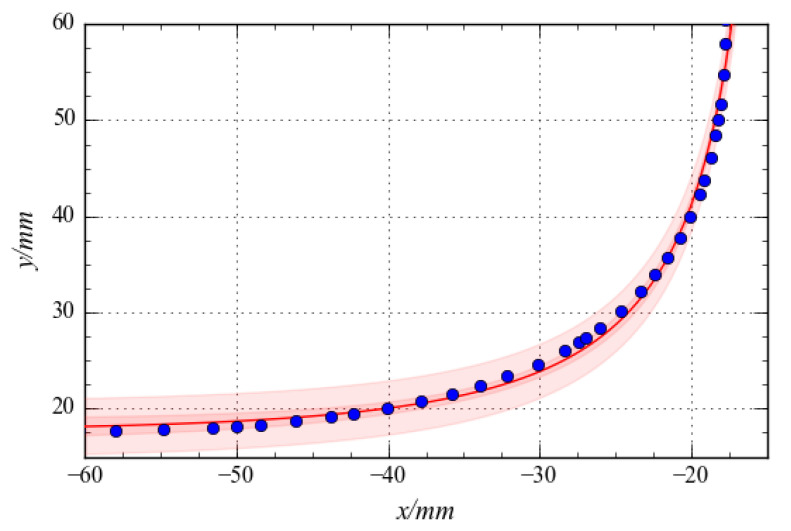
Boundary shape fitting curve.

Figure 15 depicts the spatial distribution of 31 points along the intersection of the crossarm obtained through the shape optimisation and the regression curve calculated from Equation (10). Regression curves could provide insight into the character of a specimen obtained from shape optimisation.

**Figure 16:**
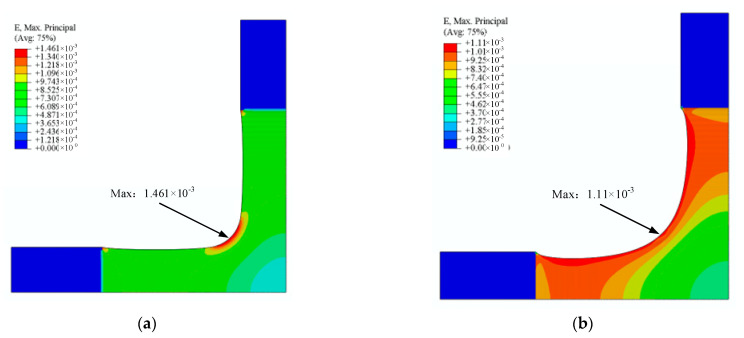
Equivalent strain distribution of specimens. (**a**) Initial specimen. (**b**) Optimised specimen.

To verify the improvement of the strain concentration, strain distributions were calculated for the original and final specimens under the same equiaxial loads (40 kN) (Figure 16). The strain of the optimised specimen at the intersection is reduced by 24.0%: the maximum strain of the original specimen is 1.461 × 10^−3^, while the maximum strain of the optimised specimen is only 1.110 × 10^−4^. In addition, the force transfer characteristics of the optimised specimen boundary are significantly improved from the overall strain distribution (Figure 16).

## 4. Discussion

We found that the mapped relationship could quantify the biaxial test system’s alignment deviations. Overall, 99.2% (54,536 of 54,976) of the quantified alignment deviations met the percentage of all quantified errors of less than 5%. The quantification of alignment deviations is consistent with the finite element model, with the fit parameters R2 and Pr exceeding 0.999. Hence, we could quantify the biaxial test system’s alignment deviations via the optimised specimen’s strain distribution.

The alignment deviations’ identification coefficients can quantify the misalignment’s contribution with the least square method (LMS). Given the similarity of strain distribution determined by alignment deviations (Figure 11, Figure 12 and Figure 13 and Table 6), subjectively and qualitatively judging how alignment deviations affect strain distribution may be incorrect. In-plane misalignments (X1U2, X1UR3, X2U2, X2UR3, Y1U1, and Y1UR3) lead to symmetrical strain distributions about 0° and 90° in the XY plane, whereas out-of-plane misalignments (X1U3, X1UR1, X2U3, X2UR1, Y1U3, and Y1UR2) lead to symmetrical strain distributions about ±45°. By analysing strain distribution symmetry, in-plane and out-of-plane misalignment in test results can be analysed qualitatively. However, due to the linear independence of 12 strain characteristics, the strain distribution—generated by any combination of 12 alignment deviations—could be linearly expressed by LSM. In engineering practice, the size of the identification coefficients may determine the adjustment order in the alignment process. Therefore, we could use the identification coefficients to quantify the contribution of each alignment deviation to the cruciform specimen’s strain distribution.

Surprisingly, shape optimisation markedly diminished the strain concentration at the cross-arm intersection. Compared with the classic structure, the maximum strain of the optimised specimen is reduced by 24% under the same boundary conditions (Figure 16). Compared with other parameter optimisation methods [19,20,21,22,23], the shape optimisation method is more practical due to breaking through geometric parameter limitations. The topological properties of shape optimisation determined that the stress concentration at the intersection could be reduced by improving the force transfer characteristics of the boundary.

The misalignment of the test system is the primary reason for the systematic error of the test data [15]. However, current methods cannot quantify alignment deviations of the in-plane biaxial test system, yielding that accuracy of test data could only be guaranteed by the precision of the test systems’ manufacturing and assembly [26,27,28,29,30,31]; the misalignment of the in-plane biaxial test systems varied from laboratory to test, incurring an extensive scatter of test data for the same material. Therefore, quantifying alignment deviations is essential for the in-plane biaxial test to gain reliable test data.

Our four alignment deviations are caused by the spatial dislocation of the upper and lower fixture axes, not the eccentric clamping load. Theoretically, when the fixture clamping is ideal, uniaxial test system alignment deviations are independent of specimen geometry. Suppose the fixture has poor clamping; as the specimen’s geometry changes with an eccentric load, the alignment deviations may change. To ensure consistency of alignment deviations in the actual test, the specimen’s geometries should match those used to quantify uniaxial alignment deviations. Strain measurement errors, manufacturing, and installation flaws affect test results, but alignment deviations are more critical. 

When using actual testing data to quantify alignment deviations, the model’s accuracy may be reduced. Finite element simulation accuracy affects the quantified model of alignment deviations. Numerical simulations do not account for manufacturing, installation, and test errors, which affect strain measurement in actual tests. Thus, controlling errors outside the finite element simulation is crucial to avoid reducing alignment test accuracy. 

These findings help us quantify alignment deviations of the in-plane test systems and design the cruciform specimen. For engineering practice, engineers could find problems in design, manufacturing, and assembly processes through quantitative analysis of the alignment deviations to enhance the test system’s accuracy and improve assembly efficiency. For material research, scholars could modify the constitutive model of materials and diminish the dispersion of material test data caused by the difference in pieces of the material test systems by quantifying the alignment deviations of the in-plan test system. To prevent wrong conclusions from being drawn due to systematic errors, the quantitative alignment deviation can be used to analyse the test error of material test results. During production and assembly, it can direct the alignment adjustment of the in-plane biaxial material testing system.

## 5. Conclusions

Our study aims to quantify alignment deviations via the strain distribution of the optimised cruciform specimen. We first mapped a model to quantify the alignment deviations of the in-plane biaxial test system via automated machine learning (AutoML). In addition, we proposed the identification coefficients to quantify the contribution of alignment deviations to the cruciform specimen’s strain distribution. Furthermore, a new boundary form for cruciform specimens has been proposed based on nonparametric shape optimisation. The quantified methods of alignment deviations may be beneficial to gaining reliable test data, improving assembly accuracy, and aid in designing cruciform specimens of the in-plane biaxial test system.

According to the current study, we have two relevant prospects for future research. First, this quantifiable approach to determining alignment errors might also be used to calibrate uniaxial, multiaxial, and other high-precision processing components of systems, as the specimen’s strain distribution directly reflects the equipment’s alignment deviations. Second, due to the lack of methods to quantify the loading coaxiality, we recommend standard makers pay more attention to quantifying the alignment deviations of multiaxial test systems.

## Figures and Tables

**Table 1:** A summary of standards and test methods.

Item	Description
ASTM E6	Terminology Relating to Methods of Mechanical Testing.
ASTM E8/E8 M-21	Standard Test Methods for Tension Testing of Metallic Materials.
ISO 16808:2014	Metallic Materials—Sheet and Strip—Determination of Biaxial Stress–Strain Curve by Means of Bulge Test with Optical Measuring Systems.
ISO 16842: 2021	Metallic materials—Sheet and Strip–Biaxial Tensile Testing Method Using a Cruciform Test Piece.
ISO 6892–1:2019	Metallic materials—Tensile Testing—Part 1: Method of Test at Room Temperature.
ISO 6892–2:2018	Metallic materials—Tensile Testing—Part 2: Method of Test at Room Temperature.
ISO 6892–3:2015	Metallic Materials—Tensile Testing—Part 3: Method of Test at Low Temperature.
ISO 6892–4:2015	Metallic Materials—Tensile Testing—Part 4: Method of Test in Liquid Helium.
ISO 12004–1:2020	Metallic materials—Determination of Forming-Limit Curves for Sheet and Strip—Part 1: Measurement and Application of Forming—Limit Diagrams in the Press Shop
ISO 12004–2:2021	Metallic materials—Determination of Forming—Limit Curves for Sheet and Strip—Part 2: Determination of Forming—Limit Curves in the Laboratory.
ASTM E2218-02	Standard Test Method for Determining Forming Limit Curves.
ASTM E 251	Test Methods for Performance Characteristics of Metallic Bonded Resistance Strain Gauges.
ASTM E 466	Practice for Conducting Force Controlled Constant Amplitude Axial Fatigue Tests of Metallic Materials.
ASTM E1012-19	Standard Practice for Verification of Testing Frame and Specimen Alignment Under Tensile and Compressive Axial Force Application
ISO 9513:2012	Metallic Materials—Calibration of Extensometer Systems Used in Uniaxial Testing.
ISO 204:2018	Metallic Materials—Uniaxial Creep Testing in Tension—Method of Test.
ISO 376:2011	Metallic Materials—Calibration of Force-Proving Instruments Used for the Verification of Uniaxial Testing Machines.
ISO 7500–1:2018	Metallic materials—Calibration and Verification of Static Uniaxial Testing Machines—Part 1: Tension/Compression Testing Machines—Calibration and Verification of the Force-Measuring System.
ISO 7500–2:2006	Metallic Materials—Verification of Static Uniaxial Testing Machines—Part 2: Tension Creep Testing Machines—Verification of the Applied Force.
ASTM D5617–04 (2015)	Standard Test Method for Multiaxial Tension Test for Geosynthetics.
ISO/TR 12112:2018	Metallic Materials—Principles and Designs for Multiaxial Fatigue Testing.
ISO 24281:2021	Textiles—Biaxial Tensile Properties of Woven Fabric—Determination of Maximum Force and Elongation at Maximum Force Using the Grab Method.
ISO 21022:2018	Test Method for Fiber-Reinforced Cementitious Composites—Load-Deflection Curve Using Circular Plates.

**Table 2:** Alignment deviations for the FEM model.

		Minimum	Maximum
X1	U2	−1.000 mm	1.000 mm
	U3	−1.000 mm	1.000 mm
	UR2	−0.012 rad	0.012 rad
	UR3	−0.012 rad	0.012 rad
X2	U2	−1.000 mm	1.000 mm
	U3	−1.000 mm	1.000 mm
	UR2	−0.012 rad	0.012 rad
	UR3	−0.012 rad	0.012 rad
Y1	U2	−1.000 mm	1.000 mm
	U3	−1.000 mm	1.000 mm
	UR2	−0.012 rad	0.012 rad
	UR3	−0.012 rad	0.012 rad

**Table 3:** Quantified error analysis of alignment deviations.

Deviations		Minimum	Maximum	Mean	Std. Deviation	N
X1	U2	−0.0170 mm	0.0217 mm	−0.000042 mm	0.002158 mm	54,976
	U3	−0.0449 mm	0.0424 mm	−0.000031 mm	0.005344 mm	54,976
	UR2	−0.0961 rad	0.1268 rad	0.000012 rad	0.007932 rad	54,976
	UR3	−0.0701 rad	0.0805 rad	0.000083 rad	0.005912 rad	54,976
X2	U2	−0.0284 mm	0.0225 mm	0.000041 mm	0.002306 mm	54,976
	U3	−0.0381 mm	0.0558 mm	−0.000045 mm	0.005104 mm	54,976
	UR2	−0.0845 rad	0.0926 rad	0.000068 rad	0.007924 rad	54,976
	UR3	−0.0809 rad	0.0995 rad	−0.000031 rad	0.005833 rad	54,976
Y1	U2	−0.0631 mm	0.0754 mm	0.000043 mm	0.004029 mm	54,976
	U3	−0.0367 mm	0.0890 mm	−0.000087 mm	0.004981 mm	54,976
	UR2	−0.5954 rad	0.1317 rad	−0.000040 rad	0.006691 rad	54,976
	UR3	−0.3167 rad	0.0529 rad	0.000060 rad	0.004048 rad	54,976

**Table 4:** Correlation analysis of the finite element model and the quantified model.

Deviation		R2	Pearson-R
X1	U2	0.999944	0.999972
	U3	0.999661	0.999832
	UR2	0.999241	0.999620
	UR3	0.999560	0.999785
X2	U2	0.999934	0.999967
	U3	0.999694	0.999847
	UR2	0.999263	0.999632
	UR3	0.999597	0.999804
Y1	U2	0.999788	0.999898
	U3	0.999684	0.999843
	UR2	0.999506	0.999755
	UR3	0.999795	0.999898

**Table 5:** Uniform distribution tests of the 12 alignment deviations.

Deviations		Uniform Parameters		Kolmogorov–Smirnov Z	Asymp. Sig. (2-Tailed)	N
		Minimum	Maximum			
X1	U2	−1.000 mm	1.000 mm	0.026	1.000	54,976
	U3	−1.000 mm	1.000 mm	0.026	1.000	54,976
	UR2	−0.012 rad	0.012 rad	0.036	1.000	54,976
	UR3	−0.012 rad	0.012 rad	0.041	1.000	54,976
X2	U2	−1.000 mm	1.000 mm	0.029	1.000	54,976
	U3	−1.000 mm	1.000 mm	0.032	1.000	54,976
	UR2	−0.012 rad	0.012 rad	0.021	1.000	54,976
	UR3	−0.012 rad	0.012 rad	0.032	1.000	54,976
Y1	U2	−1.000 mm	1.000 mm	0.026	1.000	54,976
	U3	−1.000 mm	1.000 mm	0.026	1.000	54,976
	UR2	−0.012 rad	0.012 rad	0.035	1.000	54,976
	UR3	−0.012 rad	0.01 2 rad	0.027	1.000	54,976

**Table 6:** Correlation coefficient matrix of 12 characteristic strain vectors.

Item	X1U2	X1U3	X1UR2	X1UR3	X2U2	X2U3	X2UR2	X2UR3	Y1U1	Y1U3	Y1UR1	Y1UR3
X1U2	1.000	0.005	0.001	0.677	0.143	0.005	0.007	0.005	0.164	0.001	−0.001	0.217
X1U3		1.000	−0.783	0.004	0.004	0.223	0.108	0.001	0.001	−0.409	0.297	0.003
X1UR2			1.000	0.003	0.005	−0.103	−0.008	0.003	0.000	0.299	−0.197	−0.001
X1UR3				1.000	0.000	0.001	0.003	0.000	0.296	0.000	0.000	0.296
X2U2					1.000	0.004	0.003	0.683	−0.090	0.000	0.000	−0.206
X2U3						1.000	0.791	0.004	−0.001	−0.401	0.295	0.000
X2UR2							1.000	0.003	0.000	−0.0294	0.197	0.000
X2UR3								1.000	−0.259	0.000	0.000	−0.291
Y1U1									1.000	0.000	0.000	0.661
Y1U3										1.000	−0.790	−0.001
Y1UR1											1.000	0.001
Y1UR3												1.000

**Table 7:** Effective coefficients for strain distribution on a specimen.

*e*	Initial	AD-1	AD-2	AD-3	AD-4	AD-5	AD-6	AD-7	AD-8
*e*_0_	0.00012	−0.00009	−0.00035	−0.00035	−0.00030	−0.00092	−0.00040	0.00015	−0.00036
*e*_1_	0.32452	0.00035	0.32442	0.32743	0.39915	0.42814	0.32516	0.32781	0.34263
*e*_2_	0.14594	0.15346	−0.00377	0.14399	0.17817	0.19253	0.14633	0.14804	0.14998
*e*_3_	0.06628	0.07102	0.06801	−0.00388	0.08233	0.08969	0.06822	0.06784	0.06874
*e*_4_	0.57870	0.61291	0.58587	0.58078	0.00021	0.76364	0.58150	0.58471	0.60431
*e*_5_	0.65155	0.68846	0.65936	0.65214	0.79870	0.00045	0.65187	0.65806	0.68005
*e*_6_	0.06885	0.07478	0.07097	0.06917	0.08652	0.09540	−0.00589	0.07224	0.06974
*e*_7_	0.14339	0.14953	0.14188	0.14329	0.17396	0.18451	0.14315	0.00266	0.15074
*e*_8_	−0.29023	−0.30621	−0.29470	−0.28989	−0.35558	−0.38112	−0.29075	−0.29300	0.00112
*e*_9_	−0.00053	0.00031	−0.00284	0.00052	−0.00013	−0.00060	−0.00157	−0.00059	0.00122
*e*_10_	−0.00526	−0.00619	−0.00737	−0.00764	−0.00568	−0.00517	−0.00667	−0.00292	−0.00998
*e*_11_	−0.00414	−0.00403	−0.00451	−0.00526	−0.00370	−0.00391	−0.00420	−0.00230	−0.00638
*e*_12_	0.00074	−0.00003	0.00245	−0.00054	0.00034	0.00040	0.00080	0.00060	−0.00073

**Table 8:** Fitting parameters.

	Value	Std Err	Range (95% Confidence)	DOF
*a*	17.71	0.57	16.55 to 18.88	31
*b*	−2.78	0.30	−3.40 to −2.17	31
*c*	−0.08	0.01	−0.09 to −0.07	31
